# Pioglitazone Protected against Cardiac Hypertrophy via Inhibiting AKT/GSK3*β* and MAPK Signaling Pathways

**DOI:** 10.1155/2016/9174190

**Published:** 2016-03-27

**Authors:** Wen-Ying Wei, Zhen-Guo Ma, Si-Chi Xu, Ning Zhang, Qi-Zhu Tang

**Affiliations:** ^1^Department of Cardiology, Renmin Hospital of Wuhan University, Wuhan 430060, China; ^2^Cardiovascular Research Institute, Wuhan University, Jiefang Road 238, Wuhan 430060, China

## Abstract

Peroxisome proliferator activated receptor *γ* (PPAR*γ*) has been closely involved in the process of cardiovascular diseases. This study was to investigate whether pioglitazone (PIO), a PPAR*γ* agonist, could protect against pressure overload-induced cardiac hypertrophy. Mice were orally given PIO (2.5 mg/kg) from 1 week after aortic banding and continuing for 7 weeks. The morphological examination and biochemical analysis were used to evaluate the effects of PIO. Neonatal rat ventricular cardiomyocytes were also used to verify the protection of PIO against hypertrophy in vitro. The results in our study demonstrated that PIO remarkably inhibited hypertrophic response induced by aortic banding in vivo. Besides, PIO also suppressed cardiac fibrosis in vivo. PIO treatment also inhibited the activation of protein kinase B (AKT)/glycogen synthase kinase-3*β* (GSK3*β*) and mitogen-activated protein kinase (MAPK) in the heart. In addition, PIO alleviated angiotensin II-induced hypertrophic response in vitro. In conclusion, PIO could inhibit cardiac hypertrophy via attenuation of AKT/GSK3*β* and MAPK pathways.

## 1. Introduction

Cardiac hypertrophy is characterized by the dilation of heart, the enlargement of cardiac myocytes, and the accumulation of collagen. Cardiac hypertrophy could result in ventricular arrhythmia, heart failure, and sudden cardiac death [1, 2]. Although the causes and effects of cardiac hypertrophy have been extensively investigated, the underlying molecular mechanisms of cardiac hypertrophy remain unclear. Numerous studies have implicated that activation of protein kinase B (AKT)/glycogen synthase kinase-3*β* (GSK3*β*) and mitogen-activated protein kinase (MAPK) was closely involved in the process of cardiac hypertrophy [3]. Therefore, discovering new drugs which could suppress these signaling pathways would be of great importance for the treatment of cardiac hypertrophy.

Peroxisome proliferator activated receptors (PPARs), which were named for the ability to induce hepatic peroxide proliferation in response to xenobiotic stimuli, are members of nuclear receptor superfamily [4]. There are three isotypes of PPARs: PPAR-*α*, PPAR-*β*, and PPAR-*γ* [5]. PPAR*γ* expressed predominantly in adipose tissue. PPAR*γ* was found to regulate adipogenesis and insulin sensitivity [6, 7]. Studies later showed that PPAR*γ* also expressed in rat cardiac myocytes [8] and was closely involved in the process of cardiac hypertrophy. Cardiomyocyte-specific PPAR*γ* knockout mice could induce cardiac hypertrophy [9] and activation of PPAR*γ* by the specific agonist, rosiglitazone, can inhibit cardiac hypertrophy in vivo and in vitro [10]. Pioglitazone (PIO), another PPAR*γ* agonist, also displayed protective effects in the cardiovascular diseases. Pioglitazone could inhibit atherosclerosis [11] and improved left ventricular remodeling in mice with postmyocardial infarction [12]. However, the role of PIO in cardiac hypertrophy is still unclear. Previous study indicated that the PPAR*γ* agonist suppressed GSK3*β* in colon cancer cell [13]. The activation of PPAR*γ* could result in the inhibition of extracellular signal-regulated kinase (ERK) [14]. Thus, whether PIO has antagonistic actions on these signaling pathways also still needs to be investigated.

In this study, we used an animal model of cardiac hypertrophy induced by pressure overload to determine whether PIO could protect against cardiac hypertrophy, and we also uncovered the molecular mechanisms underlying the protective effects.

## 2. Materials and Methods

All animal experiments were performed according to the guidelines for the Care and Use of Laboratory Animals published by the US National Institutes of Health (NIH Publication, revised 2011) and approved by the Animal Care and Use Committee of Renmin Hospital of Wuhan University, China.

### 2.1. Reagents

PIO was purchased from Sigma-Aldrich (CDS021593, purity > 98% determined by HPLC). Angiotensin II (Ang II, A9525) was also purchased from Sigma-Aldrich. Anti-PPAR*γ* (sc-7196) was purchased from Santa Cruz Biotechnology. The first antibodies followed were purchased from Cell Signaling Technology: anti-AKT (#4691), anti-phospho-AKT (#4060), anti-GSK3*β* (#9315), anti-phospho-GSK3*β* (#9323P), anti-ERK (#4695), anti-phospho-ERK (#4370P), anti-P38 (#9212P), and anti-phospho-P38 (#4511P). Anti-GAPDH (#ab8245) was obtained from ABCAM. Anti-*α*-actin was obtained from Millipore. The secondary antibodies were obtained from LI-COR Biosciences. All other chemicals were of analytical grade.

### 2.2. Animals and Treatments

All 60 male C57BL/6 mice (8–10-week-old; male body weight: 23.5–27.5 g) were purchased from the Institute of Laboratory Animal Science, CAMS & PUMC (Beijing, China), and housed with controlled temperature and humidity. All the mice were allowed free access to food and water under a 12 h light-dark cycle in the Cardiovascular Research Institute of Wuhan University (Wuhan, China). The animals were randomly divided to either a sham surgery or AB group, which were orally treated with or without PIO (2.5 mg/kg body weight/day) for 7 weeks, and beginning 1 week after aortic banding. The AB was performed as described previously [15, 16]. The dose of PIO was selected according to our preliminary experiment (see Figure S1 in Supplementary Material available online at http://dx.doi.org/10.1155/2016/9174190). PIO was dissolved in normal saline for in vivo experiments. Eight weeks after surgery, the animals were anesthetized with 1.5% isoflurane and subjected to echocardiographic measurements, whereafter the mice were euthanized with excessive sodium pentobarbital (200 mg/kg). Hearts were dissected and weighed to calculate the heart weight/body weight (HW/BW) and heart weight/tibia length (HW/TL) ratios in the PIO-treated and vehicle-treated mice. Collected left ventricle of heart tissues was snap-frozen in liquid nitrogen and stored in minus 80°C until further experiments.

### 2.3. Echocardiography Analysis

Echocardiography was performed on euthanized mice by using the Mylab 30CV (Esaote S.P.A., Genoa, Italy) equipped with a 10 MHz linear array ultrasound transducer, as previously described [17]. Parasternal long-axis views, short-axis views, and 2D guided M-mode images of short axis at the papillary muscle level were recorded. Left ventricular end-systolic diameter (LVSD) and end-diastolic diameter (LVDD) were measured tracing with a sweep speed of 50 mm/s.

### 2.4. Histological Analysis

The removed heart tissues were arrested with 10% KCl and fixed with 10% formalin. Then we embedded the heart in paraffin and sectioned transversely. After rehydration, the sections (4-5 *μ*m) of heart were obtained and mounted onto slides and stained with haematoxylin-eosin (HE) or picrosirius red (PSR). After staining, the cross-sectional areas of the myocytes and the average collagen volume were determined by a quantitative digital analysis system (Image-Pro Plus, version 6.0; Media Cybernetics, Bethesda, MD, USA). The sections were examined blind.

### 2.5. Western Blot Analysis

The frozen heart tissues were lysed by a RIPA buffer, which is 720 *μ*L of RIPA, 100 *μ*L of cocktail, 100 *μ*L of Phos-stop, 50 *μ*L of NaF, 20 *μ*L of PMSF, and 10 *μ*L of Na_3_VO_4_ compounded in every 1 mL of lysis buffer. The protein concentrations were subsequently measured using the BCA Protein Assay Kit (cat. number 23227; Thermo Fisher Scientific, Waltham, MA, USA). Then the protein was resolved to the 10% SDS PAGE and transferred to a PVDF membrane (cat. number IPFL00010; EMD Millipore, Billerica, MA, USA). After that, the membranes were blocked and incubated with primary antibodies and secondary antibodies. Finally, the blots were observed and analyzed using Odyssey Infrared Imaging System (LI-COR Biosciences, Lincoln, NE, USA).

### 2.6. Real-Time Polymerase Chain Reaction Analysis

The total RNA was harvested from frozen left ventricle tissues or cell lysates using TRIzol (cat. number 15596026; Invitrogen Life Technologies, Carlsbad, CA, USA). 1 *μ*g RNA of each sample was used to reverse-transcribe into cDNA using the PrimeScript RT reagent Kit (cat. number RR047Q; Takara Biotechnology (Dalian) Co., Ltd.). PCR was performed using a LightCycler 480 SYBR Green Master Mix (cat. number 04896866001; Roche Diagnostics GmbH). All primer details were provided in Table 1. The mRNA levels were normalized to GAPDH.

### 2.7. Cell Culture

The isolation of primary neonatal rat ventricular cardiomyocytes (NRVCMs) was performed according to previous study [18]. Bromodeoxyuridine (0.1 mM) was used to inhibit the growth of cardiac fibroblast. Isolated primary neonatal rat ventricular cardiomyocytes were grown in Dulbecco's Modified Eagle Medium/Nutrient Mixture F-12 Ham (DMEM/F12) 1 : 1 mixture (GIBCO, C11995). NRVCMs were provided with 10% fetal bovine serum (FBS) (GIBCO, 10099), streptomycin (100 mg/mL; GIBCO, 15140), and penicillin (100 U/mL). Then the cells were cultured in an atmosphere of 5% CO_2_ with a humidified incubator (SANYO 18M) at 37°C. The cells were seeded onto six-well culture plates or 24-well plates for 24 h with DMEM and 10% FBS, later cultured with 0.5% DMEM for another 12 h. After that, Ang II (1 *μ*mol) was added to the medium in the presence or absence of PIO (20 *μ*mol/L). The viability of neonatal rat ventricular cardiomyocytes was determined by CCK-8 in 5 independent experiments.

### 2.8. Immunofluorescence Staining

NRVCMs cultured on cover slips were pretreated with or without PIO (20 *μ*mol/L) and stimulated with 1 *μ*mol Ang II for 24 h. For staining the cells, the NRVCMs were fixed with 4% formaldehyde and permeabilized in 0.1% Triton X-100. Subsequently, the cells were stained with anti-*α*-actin (1 : 100 dilution) and incubated using Alexa Fluor 568-goat anti-mouse (Invitrogen, A11017). The cross-sectional areas were measured using Image-Pro Plus 6.0. More than 100 myocytes were outlined in each group.

### 2.9. Statistical Analysis

Data are presented as mean ± SD. Comparisons were undertaken by one-way ANOVA followed by a post hoc Tukey's test. A value of *P* < 0.05 was considered significant.

## 3. Results

### 3.1. PIO Suppressed Pressure Overload-Induced Cardiac Hypertrophy In Vivo

**Figure 1:**
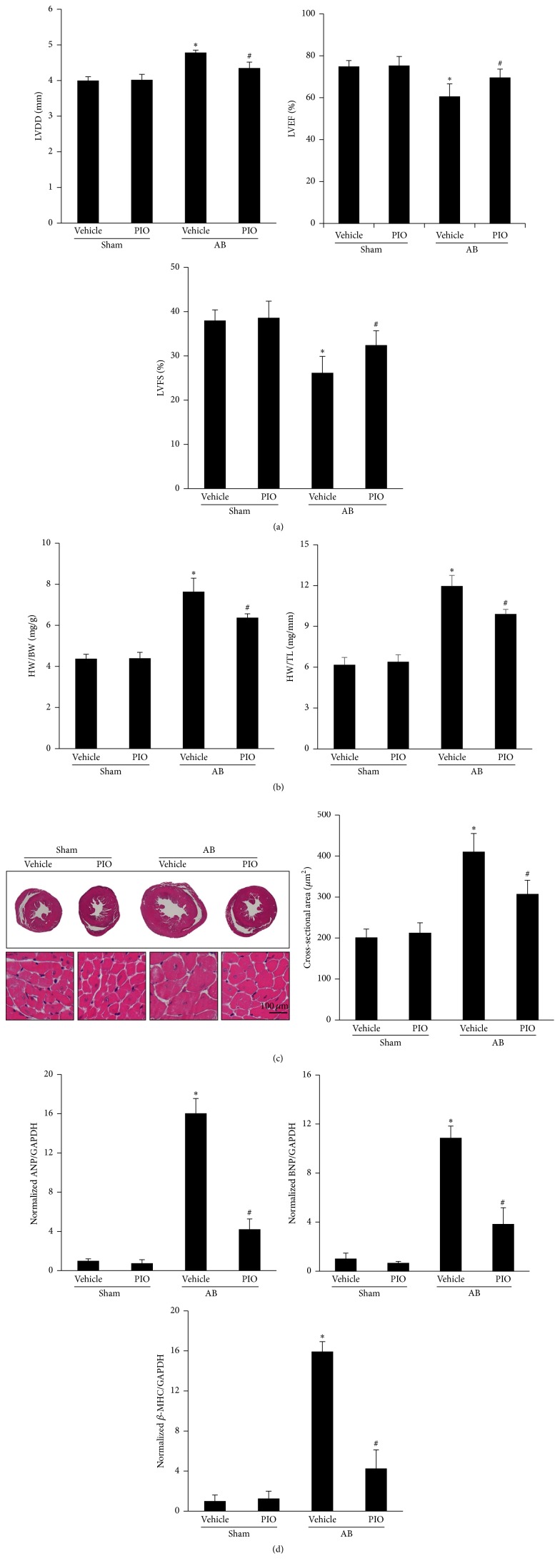
PIO inhibited cardiac hypertrophy in vivo. (a) Echocardiography results of LVDD, LVEF, and LVFS of the indicated groups (*n* = 10–12). (b) Results of the HW/BW, HW/TL ratios of the indicated groups (*n* = 13–15). (c) Gross hearts, HE staining, and cross-sectional area after 8 weeks of AB or sham with or without PIO (*n* = 4). (d) Expression levels of the transcripts for ANP, BNP, and *β*-MHC normalized by GAPDH of the indicated groups (*n* = 4). ^*∗*^ *P* < 0.05 as compared with the corresponding sham group. ^#^ *P* < 0.05 versus the AB group.

Compared with mice in sham-operated group, mice subjected to aortic banding developed a marked increase of LVDD and a decrease of heart function (Figure 1(a)). AB mice displayed a hypertrophic response as measured by the ratios of HW/BW, HW/TL, the gross heart size, and cross-sectional areas (Figures 1(b) and 1(c)). Conversely, compared with mice subjected to AB, the hypertrophic response induced by pressure overload was attenuated in mice with PIO treatment. In addition, the markers of cardiac hypertrophy, including atrial natriuretic peptide (ANP), brain natriuretic peptide (BNP), and *β*-myosin heavy chain (*β*-MHC), were also checked (Figure 1(d)). Our data demonstrated the hypertrophic markers were attenuated in AB mice treated with PIO. And no significant difference was observed in the sham-operated mice with or without PIO.

### 3.2. PIO Attenuated Cardiac Fibrosis Induced by Aortic Banding In Vivo

**Figure 2:**
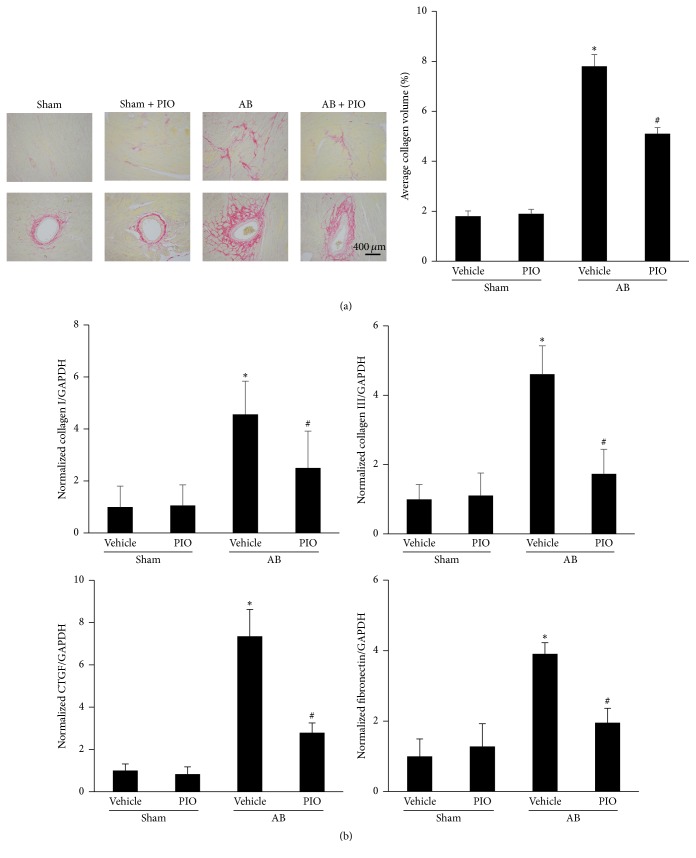
PIO attenuated cardiac fibrosis in vivo. (a) Histological sections of the left ventricle were stained for PSR and average collagen volume in group of AB or sham with or without PIO (*n* = 4). (b) Expression levels of the transcripts for collagen I, collagen III, connective tissue growth factor (CTGF), and fibronectin normalized by GAPDH of the indicated groups by real-time PCR (*n* = 4). ^*∗*^ *P* < 0.05 as compared with the corresponding sham group. ^#^ *P* < 0.05 versus the AB group.

Fibrosis, which is one of major features of cardiac hypertrophy, is characterized by the disproportionate accumulation of collagen and perivascular fibrosis [19, 20]. Thus, heart sections were stained with PSR and analyzed quantitatively to evaluate the extent of fibrosis. As shown in Figure 2(a), marked interstitial and perivascular fibrosis and increased collagen volume were observed in the mice with AB surgery, and PIO treatment could attenuate the fibrotic response. The mRNA expression levels of collagen I, collagen III, connective tissue growth factor (CTGF), and fibronectin were increased in AB group (Figure 2(b)). Though PIO cannot affect the fibrotic genes at baseline, PIO treatment decreased the increased levels of fibrotic markers induced by pressure overload.

### 3.3. PIO Protected against Cardiac Hypertrophy by Inhibiting MAPK and AKT/GSK3*β* Pathways

**Figure 3:**
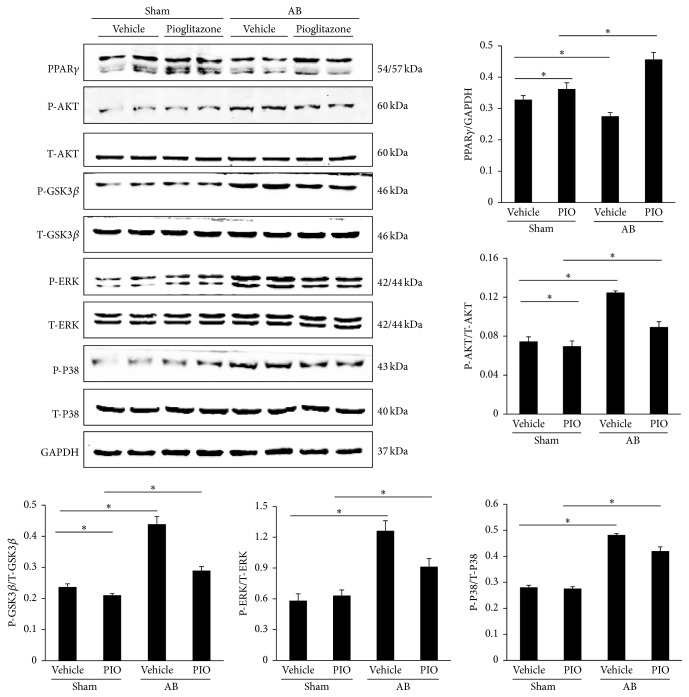
PIO protects against cardiac hypertrophy by inhibition of MAPK and AKT/GSK3*β* pathways. Representative and quantitative expression of PPAR*γ*, phosphorylated AKT, GSK3*β*, ERK, P38, and the effects of PIO in group of AB or sham with or without PIO (*n* = 6). ^*∗*^ *P* < 0.05.

Our data indicated that the levels of PPAR*γ* were decreased after long-term pressure overload. And PIO, as a PPAR*γ* agonist, apparently upregulated the expression levels of PPAR*γ* in mice given PIO with or without aortic banding (Figure 3). The MAPK and AKT/GSK3*β* signaling pathways were also checked in our study. As illustrated by Figure 3, PIO treatment alone inhibited the phosphorylated AKT and GSK3*β* in the mice without surgery. Moreover, PIO could also decrease the increased phosphorylation of AKT and GSK3*β* caused by AB. The protein levels of phosphorylated ERK and P38 were also increased after 8 weeks of aortic banding surgery, and treatment of PIO could reduce these pathways. However, there was no significant change of P-ERK and P-P38 between sham and sham + PIO group.

### 3.4. PIO Alleviated Hypertrophy of Cardiac Myocytes In Vitro

**Figure 4:**
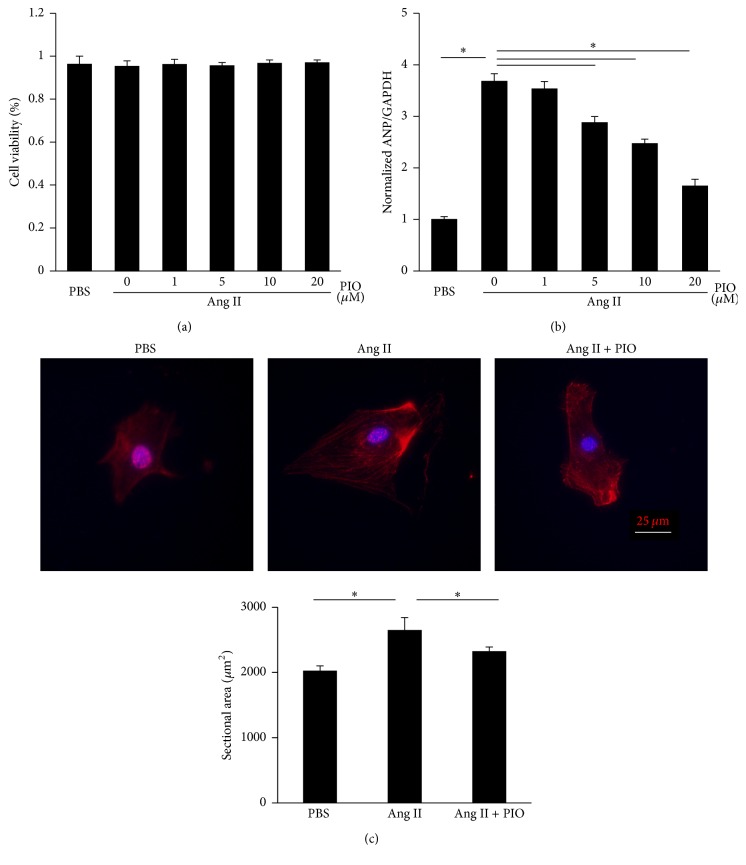
PIO alleviated hypertrophy of cardiac myocytes in vitro. (a) The effects of PIO on viability of neonatal rat ventricular cardiomyocytes were determined by CCK-8 in 5 independent experiments. (b) The levels of ANP of cardiomyocytes in indicated groups (*n* = 5). (c) The immunofluorescence of cardiomyocytes and the sectional areas of myocytes (*n* = 5). ^*∗*^ *P* < 0.05.

Taking the cardiovascular protective role of PIO into consideration, neonatal rat ventricular cardiomyocytes were used to further examine the protection of PIO against hypertrophy of myocytes in vitro. Ang II was used to induce hypertrophy in that hypertrophy induced by aortic banding was mediated partly by Ang II [21]. AKT/GSK3*β* and MAPK signaling could also be induced by Ang II [22–24]. And the treatment of PIO could not cause significant cytotoxicity in the cultured cells (Figure 4(a)). As expected, Ang II induced hypertrophy of myocytes, characterized by the increase of ANP (Figure 4(b)) and the enlargement of cross-sectional areas (Figure 4(c)). PIO treatment dose-dependently attenuated the hypertrophic response, while no significant difference was observed in baseline.

## 4. Discussion

Our study demonstrated that PIO protected against cardiac hypertrophy induced by aortic banding and inhibited hypertrophy of myocytes stimulated by Ang II. Moreover, it was observed that PIO attenuated the increases of P-AKT, P-GSK3*β*, P-ERK, and P-P38, induced by AB. Besides, PIO could attenuate the fibrosis caused by long-time pressure overload. These findings indicated that PIO played a prominent role in the protection of cardiac hypertrophy by inhibiting AKT/GSK3*β* and MAPK signaling pathways.

A number of published studies implicated that a wide array of intracellular signaling pathways were involved in the hypertrophic response. AKT is a crucial regulator of cardiac hypertrophy [25]. Activated AKT phosphorylates and inactivates the downstream GSK3*β* signaling, further promoting the development of pathological cardiac hypertrophy [26]. Previous study indicated that long-term activation of AKT could accelerate the process of heart failure [27]. Cardiac-specific overexpression of constitutive activated AKT mutant could lead to decompensation of hypertrophy; conversely, AKT1 knockout mice were resistant to cardiac hypertrophy [28]. The data from our previous studies demonstrated that inhibition of AKT/GSK3*β* alleviated AB-induced cardiac hypertrophy [29, 30]. Consistent with these results, we also found that PIO treatment reduced the activation of AKT/GSK3*β* pathways, accompanied with the increase of PPAR*γ*. The potential mechanism underlying downregulation of AKT signaling caused by PIO may be correlated with PPAR*γ*-ligand-mediated upregulation of PTEN. And PTEN could restrain activation of AKT by dephosphorylating inositol phospholipid intermediates of the PI3K pathway [31]. In addition, PPAR*γ* attenuated AKT in a PPAR-*γ*-independent manner [32]. Future study using the specific inhibitor of PPAR*γ* will be of interest.

ERK and P38, as the members of MAPK signaling pathways, participate in gene expression associated with cardiac hypertrophy. Previous studies indicated that ERK could be activated in cardiac myocytes in response to hypertrophic stimulus, and the blockade or deletion of cardiac ERK aggravated the development of cardiac hypertrophy [33]. P38 was also activated during the pressure overload-induced-hypertrophy in vivo. And overexpression of P38 in cultured cardiomyocytes by recombinant adenoviruses caused characteristic hypertrophic responses [34]. Ji et al. found that pioglitazone could inhibit microglia inflammation by blocking p38 signaling pathways [35]. Moon et al. demonstrated that alpha-eleostearic acid, as an agonist of PPAR*γ*, could play a role of anticancer by activating PPAR*γ* and downregulating the phosphorylation of ERK [36]. Li et al. verified that pioglitazone attenuated ERK phosphorylation through stimulation of adiponectin levels [37]. Consistent with these findings, our study also found that PIO reduced the phosphorylation levels of ERK and P38 in mice subjected to aortic banding. Inconsistent with our data, previous studies demonstrated that PIO could protect against ischemia-reperfusion injury via upregulation of ERK [38]. Therefore, substantial work is needed for the precise clarification of the cardiovascular protection of PIO.

Inconsistent with our study, agonist of PPAR*γ* induced cardiac hypertrophy in both the wild type mice and cardiomyocyte-specific PPAR*γ* knockout mice, implying that the in vivo effects of the agonist on the heart are mediated by non-PPAR effects [9]. Indeed, this prohypertrophic effect does not occur in humans for the limited doses [39, 40]. In view of that high level of PPAR*γ* in the heart increased uptake of fatty acid and glucose [41], a relatively small dose of PIO was used in our study, which may explain the inconsistent results.

Fibrosis was a pathophysiologic process of cardiac hypertrophy, which is characterized by the accumulation of collagen and the increase of the extracellular matrix [42]. Koitabashi et al. found that cardiac hypertrophy was accompanied by increased level of CTGF [43]. Morais et al. indicated that fibronectin contributed to maladaptive cardiac hypertrophy [44]. It was reported that rosiglitazone could inhibit Ang II-induced CTGF expression in vascular smooth muscle cells. In this study, we also observed that the increased collagen volume and the molecular markers, including collagen I, collagen III, CTGF, and fibronectin, were alleviated after PIO treatment. Previous studies demonstrated that myocardial interstitial fibrosis induced by pressure overload may be mediated through MAPK and AKT/GSK3*β* pathways [28, 29]. The suppression of AKT/GSK3*β* and MAPK pathways caused by PIO may be the potential mechanism that mediated the antifibrotic effects.

In conclusion, the results of the present study demonstrated that PIO protected against cardiac hypertrophy in vivo and inhibited myocyte hypertrophy in vitro. And the cardioprotective effects were mediated by the inhibition of MAPK and AKT/GSK3*β* pathways. Our study provides theoretical evidence for treating cardiac hypertrophy with PIO in the clinical application.

## Supplementary Material

Effects of PIO on cardiac hypertrophy. Mice subjected to AB were given PIO (1mg/kg, 2.5 mg/kg or 5mg/kg) treatment for 3 weeks. Statistical results of heart weight (HW)/ body weight (BW) in the indicated groups (n=5).

## Figures and Tables

**Table 1:** Primers used in the study.

Gene	Species		Sequence (5′-3′)
GAPDH	Mouse	Forward	ACTCCACTCACGGCAAATTC
		Reverse	TCTCCATGGTGGTGAAGACA

GAPDH	Rat	Forward	GACATGCCGCCTGGAGAAAC
		Reverse	AGCCCAGGATGCCCTTTAGT

ANP	Mouse	Forward	ACCTGCTAGACCACCTGGAG
		Reverse	CCTTGGCTGTTATCTTCGGTACCGG

ANP	Rat	Forward	AAAGCAAACTGAGGGCTCTGCTCG
		Reverse	TTCGGTACCGGAAGCTGTTGCA

*β*-MHC	Mouse	Forward	CCGAGTCCCAGGTCAACAA
		Reverse	CTTCACGGGCACCCTTGGA

BNP	Mouse	Forward	GAGGTCACTCCTATCCTCTGG
		Reverse	GCCATTTCCTCCGACTTTTCTC

Collagen I	Mouse	Forward	TGGTACATCAGCCCGAAC
		Reverse	GTCAGCTGGATAGCGACA

Collagen III	Mouse	Forward	GTCAGCTGGATAGCGACA
		Reverse	GAAGCACAGGAGCAGGTGTAGA

CTGF	Mouse	Forward	TGTGTGATGAGCCCAAGGAC
		Reverse	AGTTGGCTCGCATCATAGTTG

Fibronectin	Mouse	Forward	CCGGTGGCTGTCAGTCA GA
		Reverse	CCGTTCCCACTGCTGATTTATC
