# Supplementary material for: Pioglitazone Protected against Cardiac Hypertrophy via Inhibiting AKT/GSK3β and MAPK Signaling Pathways
Source: PPAR Res. 2016 Mar 27;2016:9174190. doi: 10.1155/2016/9174190 (PMC4826695; doi:10.1155/2016/9174190)
Supplement: Supplementary file 1 — Effects of PIO on cardiac hypertrophy. Mice subjected to AB were given PIO (1mg/kg, 2.5 mg/kg or 5mg/kg) treatment for 3 weeks. Statistical results of heart weight (HW)/ body weight (BW) in the indicated groups (n=5). [file 9174190.f1.pdf]

Pioglitazone protected against cardiac hypertrophy via inhibiting AKT/GSK-3β and MAPK signaling pathways

Wen-Ying Wei*, Zhen-Guo Ma*, Si-Chi Xu, Ning Zhang, Qi-Zhu Tang

Department of Cardiology, Renmin Hospital of Wuhan University, Wuhan, China
Cardiovascular Research Institute of Wuhan University, Wuhan, China

Corresponding author: Qi-Zhu Tang, Department of Cardiology, Renmin Hospital of Wuhan University; Cardiovascular Research Institute, Wuhan University at Jiefang Road 238, Wuhan 430060, China. E-mail: qztang@whu.edu.cn

*These authors contributed equally to this work.

Short title: Pioglitazone against cardiac hypertrophy

SUPPLEMENTAL FIGURES AND FIGURE LEGENDS:

Supplemental Figure S1

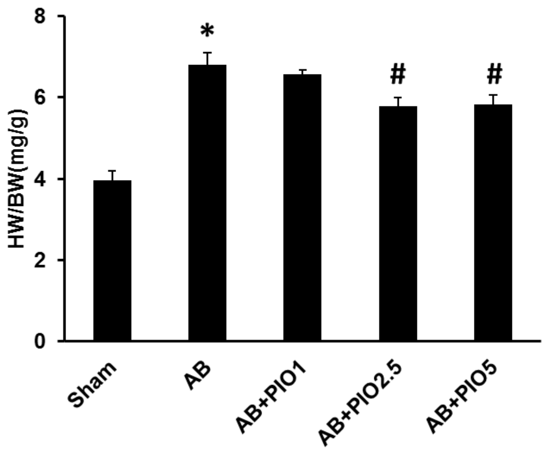

Figure S1. Effects of PIO on cardiac hypertrophy. Mice subjected to AB were given PIO (1mg/kg, 2.5 mg/kg or 5mg/kg) treatment for 3 weeks. Statistical results of heart weight (HW)/ body weight (BW) in the indicated groups (n=5).
